# ﻿Four new species of *Marasmius* subgenus *Globulares* (Marasmiaceae, Agaricales) from subtropical regions of China

**DOI:** 10.3897/mycokeys.120.157997

**Published:** 2025-08-15

**Authors:** Hong Chen, Yu-Qin Xu, Hui Zeng, Ya-Ping Hu, Sheng-Nan Wang, Jun-Qing Yan

**Affiliations:** 1 Jiangxi Provincial Key Laboratory of Excavation and Utilization of Agricultural Microorganisms, Jiangxi Agricultural University, Nanchang 330045, China Jiangxi Agricultural University Nanchang China; 2 Institute of Edible mushroom, Fujian Academy of Agricultural Sciences, Fuzhou 350011, China Fujian Academy of Agricultural Sciences Fuzhou China; 3 Nanjing Institute of Environmental Sciences, Ministry of Ecology and Environment Mountains, Nanjing 210042, China Ministry of Ecology and Environment Mountains Nanjing China

**Keywords:** Basidiomycetes, new taxa, phylogeny, taxonomy

## Abstract

*Marasmius* is a large genus in Agaricales, exhibiting rich species diversity and a wide distribution. In this study, four new species of Marasmius belonging to the subgenus Globulares were identified in subtropical regions of China. Morphologically, *M.blandus* is characterized by a light orange pileus, with a non-striate surface, medium-sized basidiospores, and subfusiform to narrowly utriform pleurocystidia; *M.xingshanensis* is identified by a brown pileus, variably shaped pleurocystidia, and absence of cheilocystidia; *M.vulgaris* is identified by large basidiospores, which are up to 16.0 μm long, narrowly fusiform to lageniform pleurocystidia, often with capitate, papillate, or constricted moniliform structures at apices; *M.subpurpureostriatus* is recognized by a grayish-green pileus with deeply sulcate violet striae, lamellae distant, and clavate to fusoid-clavate basidiospores, which are up to 22.0 μm long. The distinct taxonomic status of these four new species was confirmed by their positions in the 4-locus (ITS, LSU, *tef-1α*, *rpb2*) phylogenetic trees. Detailed descriptions and morphological photographs of four new species are provided in this paper.

## ﻿Introduction

*Marasmius* Fr., with *M.rotula* (Scop.) Fr. designated as the type species, was established by Fries (1835). According to the Index Fungorum, the genus contains 2,083 records and comprises approximately 700 species (Oliveira et al. 2024). It is characterized by the small to medium-sized basidiomata with convex to campanulate pilei; adnate to adnexed, or free lamellae that may be collariate; an insititious or non-insititious stipe; white spore print; inamyloid, smooth, hyaline, thin-walled basidiospores; a hymeniform pileipellis. It typically grows on soil, dead branches, or leaf litter in forests and is distributed worldwide, with high diversity in tropical and subtropical areas (Retnowati and Desjardin 2022).

Singer classified *Marasmius* into twelve sections based on cortical structure and spore characteristics (Singer 1958; Singer 1976; Singer 1986). This classification was widely accepted until the end of the last century. Recent molecular phylogenetic studies have shown that *Marasmius* is polyphyletic (Wilson and Desjardin 2005). By analyzing nrITS and nLSU rDNA sequences, Wilson and Desjardin (2005), Wannathes et al. (2009a), Tan et al. (2009), and Jenkinson et al. (2014) restricted the genus to a monophyletic lineage containing only the five sections defined by Singer, including the sections *Marasmius*, *Globulares*, *Sicci*, *Leveilleani* and *Neosessiles*. Other sections recognized by Singer are currently excluded from *Marasmius*. Subsequently, Oliveira et al. (2024) proposed two subgenera – *Marasmius* and *Globulares*, based on morphological and phylogenetic analysis of multilocus (SSU, LSU, ITS, *rpb2* and *tef*-*1α*). The subgenus Marasmius is characterized by thin basidiomata with an insititious stipe, inamyloid or dextrinoid trama, and includes sect. Crinis-eques, sect. Marasmius, sect. Sanguirotales, sect. Variabilispori, and sect. Sicciformes. The subgenus Globulares is characterized by a great variety of basidiomata, stipe with a basal mycelium (non-insititious), exclusively dextrinoid trama, and includes sect. Globulares and sect. Sicci.

Macrofungal species diversity in China is remarkably rich, with the subtropical regions emerging as hotspots for biodiversity research due to their complex topography and climatic conditions. In recent years, at least thirteen new species within *Marasmius* have been discovered in China (Deng and Li 2011; Deng et al. 2011; Deng et al. 2012; Yang et al. 2013; Deng et al. 2015; Wang et al. 2015; Deng et al. 2017; Liang et al. 2017; Li et al. 2023; Zhang et al. 2023). During biodiversity surveys in China’s subtropical regions, we discovered four new species within the subgenus Globulares. This study provides a detailed elucidation of the newly discovered species through morphological comparisons and phylogenetic analyses​.

## ﻿Materials and methods

### ﻿Morphological studies

Specimens were collected from Hubei, Jiangxi, and Zhejiang provinces of China between 2019 and 2024 and were deposited in the Herbarium of Fungi, Jiangxi Agricultural University (HFJAU). Macroscopic characteristics were recorded from fresh specimens. Color codes are from Kornerup and Wanscher (1978). Microscopic features were described from dried material mounted in H_2_O, 5% aqueous KOH, Melzer’s reagent and Congo Red using an Olympus BX-53 microscope (Olympus Corporation, Tokyo, Japan) (Horak 2005). For each collection, at least 40 basidiospores, basidia, and cystidia were measured. The range of spore size is expressed in the form (a) b–c (d), in which “a” and “d” represent the minimum and maximum values; 90% of the spores fall within the range “b–c”. The meanings of the other spore characteristics are as follows: “Q” stands for the ratio of length to width (Chen et al. 2024; Yan et al. 2024).

### ﻿DNA extraction, PCR ampliﬁcation, and sequencing

Genomic DNA was extracted from dried specimens using the NuClean Plant Genomic DNA kit (CWBIO, China) (Hopple and Vilgalys 1999; Wang et al. 2022). The ITS, LSU, *tef-1α*, and *rpb2* regions were amplified using the respective primer pairs of ITS1F/ITS4, LR0R/LR7, EF983F/EF1567R, and rpb2-6F/rpb2-7.1R (Hopple and Vilgalys 1999; Sánchez‐Ramírez et al. 2014).

PCR amplification was conducted in a 25 µL reaction system as follows: 1 µL DNA, 2 µL primers, 9.5 µL ddH_2_O, and 12.5 µL 2× Taq Master Mix (Dye Plus). For ITS, PCR was carried out using a touchdown amplification procedure: initial 95 °C for 5 min, and then 14 cycles of denaturing at 95 °C for 30 s, annealing at 65 °C for 45 s (−1 °C per cycle), extension at 72 °C for 1 min, and then 30 cycles of denaturing at 95 °C for 30 s, annealing at 52 °C for 30 s, and extension at 72 °C for 1 min, with the final extension at 72 °C for 10 min (Yan et al. 2022). For the other regions, the procedure was initial 98 °C for 5 min, and then 8 cycles of denaturing at 98 °C for 5 s, annealing at 61 °C for 40 s (−1 °C per cycle), extension at 72 °C for 2 min, and then 35 cycles of denaturing at 98 °C for 5 s, annealing at 54 °C for 1.5 min, extension at 72 °C for 2 min, with the final extension at 72 °C for 10 min (Sánchez‐Ramírez et al. 2014; Chen et al. 2024). The PCR products were sequenced by Qing Ke Biotechnology Co. Ltd. (Wuhan City, China).

### ﻿Alignment and phylogenetic analyses

Sequence reads were assembled and edited using Sequencher v.5.4 and were deposited in GenBank (GB) database. Based on the research by Oliveira et al. (2024), and the similarity of these new species to the most closely related sequences identified in the BLAST results of ITS, a total of 187 sequences, including 78 ITS, 55 LSU, 27 *tef-1α* and 27 *rpb2* regions, were used in subsequent analyses. Four species of Marasmiussubg.Marasmius were designated as outgroups. Details are presented in Table 1.

**Table 1.** Details of sequences used in the phylogenetic analyses.

Species	Voucher	ITS	LSU	*tef-1α*	*rpb2*	Reference
*Marasmiusalbopurpureus*	GDGM57089	KP127675				Wang et al. (2015)
*M.albopurpureus*	GDGM57201(holotype)	KP127674	KP127676			Wang et al. (2015)
*M.altoribeirensis*	JO532	KP635204	KP635158	PP026056	OR896487	Oliveira et al. (2022)
*M.auranticapitatus*	MC4554(holotype)	ON502670	ON502734			Oliveira et al. (2022)
*M.avellaneus*	FK1616(holotype)	MN714031	OR656956	PP026134	OR896412	Oliveira et al. (2020a)
*M.bekolacongoli*	Lockwood2131638	KX148982				Shay et al. (2017)
***M.blandus***	**HFJAU2362(holotype)**	**PV363543**	**PV363573**	**PV390072**	**PV394691**	this study
***M.blandus***	**HFJAU3367**	**PV363545**			**PV394694**	this study
***M.blandus***	**HFJAU4946**	**PV363544**	**PV363576**		**PV394696**	this study
***M.blandus***	**HFJAU5635**	**PV363546**	**PV363580**	**PV390077**	**PV394698**	this study
*M.bondoi*	NW390	EU935477				Wannathes et al. (2009a)
*M.bondoi*	NW386(holotype)	EU935476				Wannathes et al. (2009a)
*M.brunneospermus*	KPM-NC 0005011(holotype)	FJ904969	FJ904951			Antonín et al. (2010a)
*M.campestris*	HKAS 80857(holotype)	KJ126766	KJ126768			Liang et al. (2017)
*M.campestris*	HKAS 80858	KJ126767	KJ126769			Antonín (2003)
*M.castanocephalus*	JO523(holotype)	ON502679	ON502746	ON553955	ON553943	Oliveira et al. (2022)
M.cohaerensvar.cohaerens	BRNM652833	GU266261	GU266268			Antonín et al. (2010b)
M.cohaerensvar.cohaerens	BRNM695761	GU266260	GU266267			Antonín et al. (2010b)
M.cohaerensvar.lachnophyllus	DED4071	OR636637	OR656962			Oliveira et al. (2024)
M.cohaerensvar.mandshuricus	LE 295991	KF774167	KF896247			Kiyashko et al. (2014)
M.cohaerensvar.mandshuricus	LE 295986(holotype)	KF774171	KF896245			Kiyashko et al. (2014)
M.confertusvar.tenuicystidiatus	BRNM 718808	HQ607374	HQ607375			Antonín et al. (2011)
M.confertusvar.tenuicystidiatus	HMAS 293268	OR236975				Antonín et al. (2011)
*M.cystidiatus*	DED4594	OR636641	OR656966	PP026074		Oliveira et al. (2024)
*M.decipiens*	DED3612	OR636642	OR656967			Oliveira et al. (2024)
*M.decipiens*	DED4146	OR636643	OR656968			Oliveira et al. (2024)
*M.dimorphus*	JO298	KP635174	KP635129	PP026065	OR896448	Oliveira et al. (2020b)
*M.dimorphus*	JO334	KP635175	KP635130	PP026066		Oliveira et al. (2020b)
*M.floridanus*	DED5164	OR636645	OR656973			Oliveira et al. (2024)
*M.fusicystidiosus*	BRNM 714567(holotype)	FJ917624	FJ936144			Antonín et al. (2010a)
*M.galbinus*	GDGM27251(holotype)	HQ709445				Deng and Li (2011)
*M.gracilis*	JO90(holotype)	MN714038	OR656976	PP026146	OR896407	Oliveira et al. (2020a)
*M.graminipes*	NW078(holotype)	EU935479				Wannathes et al. (2009a)
*M.grandiviridis*	NW152(holotype)	OR636648	OR656977			Wannathes et al. (2009a)
*M.haematocephalus*	JO533	ON502673	ON502729			Oliveira et al. (2022)
*M.indopurpureostriatu*	KD 14-001(holotype)	KT004442				Dutta et al. (2015)
*M.macrocystidiosus*	KP-13	HF546218				Kiyashko et al. (2014)
*M.macrocystidiosus*	LE 295996(holotype)	KF774136	KF896246			Kiyashko et al. (2014)
*M.magnus*	AC 1001(holotype)	KX228846				Magnago et al. (2016)
*M.magnus*	NP 514	KX228850				Magnago et al. (2016)
*M.maximus*	BRNM 714571	FJ904977	FJ904958			Antonín et al. (2010b)
*M.maximus*	BRNM 714671	FJ904975	FJ904957			Antonín et al. (2010b)
*M.mokfaensis*	DED7726(holotype)	EU643516				Wannathes et al. (2009b)
*M.nigrodiscus*	TENN 59556	KF774139				Kiyashko et al. (2014)
*M.nigrodiscus*	DED5163	OR636667	OR657005	PP026062		Oliveira et al. (2024)
*M.nivicola*	BRNM 714573	FJ904971	FJ904953			Antonín et al. (2010b)
*M.nivicola*	KPM-NC 0006038 (holotype)	FJ904973	FJ904955			Antonín et al. (2010b)
*M.odoratus*	CAL:1264(holotype)	KT180332				Farook and Manimohan (2015)
*M.oreades*	NN055694	JN943604	JN941144		JQ031099	Kotowski et al. (2019)
*M.oreades*	ZRL2015086	LT716048	KY418864	KY419066	KY419010	unpublished
*M.pinicola*	Li20220706-13	OQ941785				Li et al. (2023)
*M.pinicola*	Li20220706-15(holotype)	OQ941786				Li et al. (2023)
*M.pseudoconfertus*	HKAS 49088(holotype)	HQ832733				Deng et al. (2011)
*M.pseudopurpureostriatus*	NW286(holotype)	OR636672	OR657016			Wannathes et al. (2009a)
*M.puerariae*	R Kirschner & C-J Chen 2139 (type)	JX470333	JX470332			Kirschner et al. (2013)
*M.purpureostriatus*	NW158	EU935539				Wannathes et al. (2009a)
*M.rhabarbarinus*	JO494	KP635192	KP635147	PP026070	OR896451	Oliveira et al. (2020b)
*M.rhabarbarinus*	JO457	KP635191	KP635146	PP026069	OR896450	Oliveira et al. (2020b)
*M.rhabarbarinoides*	JO66	KP635190	KP635145	PP026068		Oliveira et al. (2020b)
*M.roseus*	JO352(holotype)	ON502678	ON502745	ON553954	ON553944	Oliveira et al. (2022)
*M.rotula*	AW274	MN714042	OR657020	PP026113	OR896415	Oliveira et al. (2020a)
*M.rubicundus*	JO464(holotype)	ON502658	ON502728	ON553948	ON553937	Oliveira et al. (2022)
*M.silvicola*	JO357	KP635194	KP635149	PP026060		Oliveira et al. (2024)
*M.silvicola*	JO362	KP635195			OR896485	Oliveira et al. (2020b)
*M.silvicola*	JO366	KP635196	KP635150	PP026061	OR896486	Oliveira et al. (2020b)
*M.spegazzinii*	JO467	KP635197	KP635151	PP026075	OR896488	Wannathes et al. (2009a)
***M.subpurpureostriatus***	**HFJAU4740(holotype)**	**PV363556**	**PV363575**	**PV390073**	**PV394695**	this study
*M.subvigintifolius*	JO242(holotype)	MN714035	OR657031	PP026132	OR896411	Oliveira et al. (2020a)
***M.vulgaris***	**HFJAU0904**	**PV363550**			**PV394688**	this study
***M.vulgaris***	**HFJAU1901**	**PV363551**	**PV363571**	**PV390070**	**PV394689**	this study
***M.vulgaris***	**HFJAU1976**	**PV363552**	**PV363572**	**PV390071**	**PV394690**	this study
***M.vulgaris***	**HFJAU2719**	**PV363553**				this study
***M.vulgaris***	**HFJAU2748**	**PV363554**			**PV394692**	this study
***M.vulgaris***	**HFJAU2875(holotype)**	**PV363555**	**PV363574**		**PV394693**	this study
*M.wynneae*	HCCN-G86	FJ904979	FJ904961			Antonín et al. (2010b)
***M.xingshanensis***	**HFJAU5344**	**PV363547**	**PV363577**	**PV390074**		this study
***M.xingshanensis***	**HFJAU5540**	**PV363548**	**PV363578**	**PV390075**		this study
***M.xingshanensis***	**HFJAU5544(holotype)**	**PV363549**	**PV363579**	**PV390076**	**PV394697**	this study

**Figure 1.**
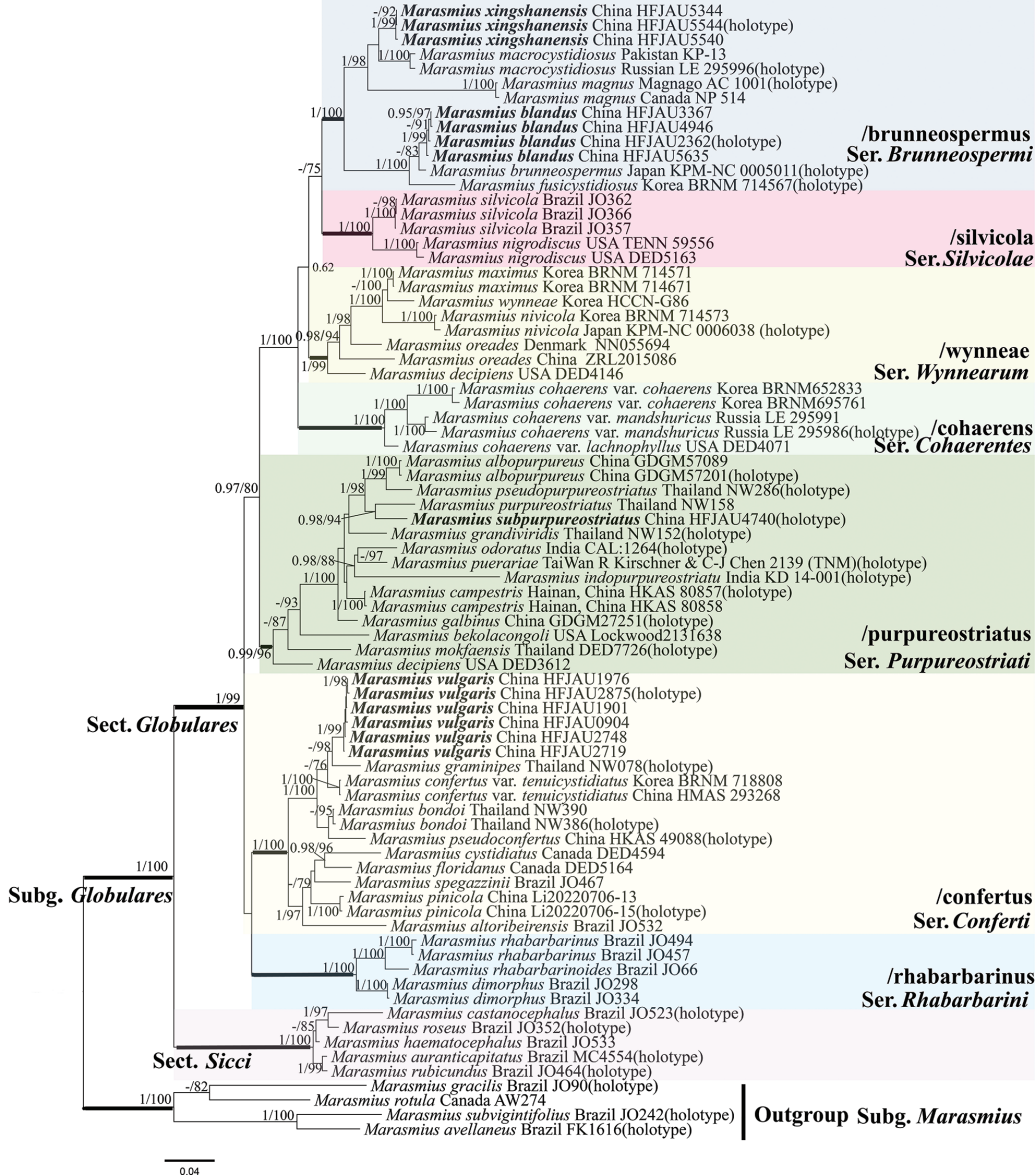
Phylogram of *Marasmius* generated by Bayesian inference (BI) analysis based on sequences of ITS + LSU + *tef-1α* + *rpb2*. It was rooted with *M.avellaneus*, *M.gracilis*, *M.rotula* and *M.subvigintifolius*. Bayesian inference (BI-PP) ≥ 0.95 and ML bootstrap proportions (ML-BP) ≥ 75 are indicated as PP/BP. The new taxa are marked in bold.

Sequence datasets, containing intron regions, were separately aligned using MAFFT v.7 (Katoh and Standley 2013). BI and ML phylogenetic analyses of the processed sequences were run using Mrbayes v.3.2.7a and IQ-TREE v.2.1.2, respectively (Ronquist and Huelsenbeck 2003; Nguyen et al. 2014). The best-fit models for BI were determined by PartitionFinder, complying with Corrected Akaike information criterion (AICc) (Lanfear et al. 2017). For the ML analysis, 10000 replicates are performed based on the ultrafast bootstrap option of ML that allowed partitions from different seeds. For the BI analysis, the Markov chains were run for 1 million generations. The ﬁrst 25% of trees were discarded as burn-in. Branches with Bayesian posterior probability (BI-PP) ≥ 0.95 and ML bootstrap support (ML-BP) ≥ 75% are shown in the tree (Fig. 1). The designation of the clade names is based on the study by Oliveira et al. (2024).

## ﻿Results

### ﻿Phylogenetic analysis

A total of 4086 characters from 78 taxa were used in phylogenetic analyses (ITS 931 bp; LSU 1328 bp; *tef-1α* 1081 bp; *rpb2* 746 bp), of which 2673 were constant, 1135 were parsimony-informative, and 278 were singleton. The best-ﬁt models of ML: HKY + F + G4 for ITS, TIM3e + I + G4 for LSU, TIM2e + I + G4 for *tef-1α* and *rpb2*. The best-ﬁt models of BI: HKY + F + G4 for ITS, SYM + I + G4 for LSU, SYM + I + G4 for *tef-1α* and *rpb2*. For Bayes analysis, the average standard deviation of split frequencies less than 0.01 after 610000 generations.

The results of the phylogenetic analysis are shown in Fig. 1. Four new species respectively formed distinct and stable branches. *Marasmiusblandus* belongs to /brunneospermus clade and groups together with *M.brunneospermus* Har. Takah. and *M.fusicystidiosus* Antonín, Ryoo & H.D. Shin (BI-PP = 1, ML-BP = 100%). *Marasmiusxingshanensis* belongs to /brunneospermus clade and groups together with *M.macrocystidiosus* Kiyashko & E.F. Malysheva. and *M.magnus* A.C. Magnago & J.S. Oliveira (BI-PP = 1, ML-BP = 98%). *Marasmiusvulgaris* belongs to /confertus clade and is closely related to *M.graminipes* Wannathes, Desjardin & Lumyong, but this grouping has unstable support in Bayesian analysis. *Marasmiussubpurpureostriatus* belongs to /purpureostriatus clade and is closely related to *M.purpureostriatus* Hongo.

### ﻿Taxonomy

#### ﻿ Marasmius blandus

Taxon classificationFungiAgaricalesMarasmiaceae

J.Q. Yan & H. Chen sp. nov.

E10106AB-2542-5C5D-BA86-EF852375DCEC

858718

Fig. 2

##### Etymology.

“blandus” refers to its smooth and non-striate pileus.

**Figure 2.**
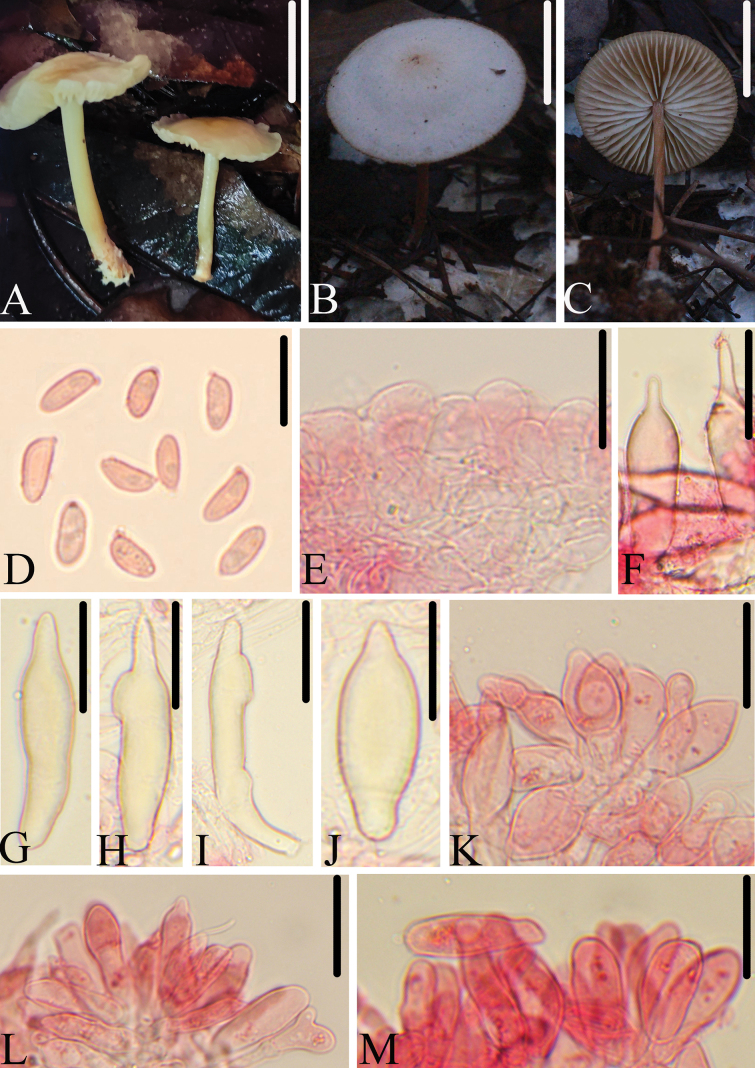
Morphological structures of *M.blandus*. A–C. Basidiomata; D. Basidiospores; E. Pileipellis; F–J. Pleurocystidia; K–M. Cheilocystidia. All microscopic structures were observed in 5% KOH, and used 1% Congo red. Scale bars: 20 mm (A–C); 10 μm (D); 30 μm (E–J); 20 μm (K–M).

##### Holotype.

China • JiangXi Province, Jiangxi Agricultural University, 6 October 2020, collected by Jia-Yue Sun, HFJAU2362.

##### Diagnosis.

*Marasmiusblandus* is mainly characterized by the rather small basidiomata, light orange pileus, with a non-striate surface; basidiospores mainly shorter than 7.5 μm; variably shaped pleurocystidia, subfusiform, narrowly utriform, with a short or long papilla at the apex and rarely with nodulose on the surface; cheilocystidia clavate, subfusiform, apex obtuse, rarely with short papilla or branched. It differs from *M.brunneospermus* by having smaller cheilocystidia, which are mainly shorter than 30 μm in length.

##### Description.

Pileus 25–50 mm, plano-convex to plane, with or without a slight obtuse umbo at the center, smooth, non-striate, hygrophanous, light orange (6A5–6) at center, slightly paler to white towards margin, drying out to white, the center and the margin with pale brown. Context thin, white. Lamellae 3.0–5.0 mm broad, adnexed, ventricose, subdistant, white with slightly brown, with 2–3 tiers of lamellules, edges even, concolorous. Stipe 20–50 mm long, 2.0–3.0 mm thick, central, cylindric, equal, fibrous, hollow, light yellow (4A3–4), becoming reddish brown (8D5–6) as stipe dries, smooth, apex velutinous, and the base covered with white mycelium.

Basidiospores (5.0)5.5–7.5(8.0) × 2.4–3.5 μm (av = 6.5 × 3.0 μm), Q = (1.6)1.8–2.6(3.0), elongated-ellipsoid to cylindrical, slightly flattened on one side in profile, 2.4–4.0 μm broad, elongated-ellipsoid to cylindrical in face view, smooth, colorless, hyaline, inamyloid, thin-walled. Basidia 24.0–30.5 × 4.0–6.5 μm, clavate, 4-spored. Pleurocystidia 37.0–80.0 × 7.5–18.0 μm, variable, subfusiform, narrowly utriform, apex with short or long papilla, surface rarely with nodulose, smooth, slightly thick-walled, yellowish in 5% KOH. Cheilocystidia 18.5–31.0(38.0) × 5.0–15.5 μm, variable, clavate, subfusiform, apex obtuse, rarely with short papilla or branched, smooth, thin-walled. Pileipellis a hymeniderm composed of cells 16.5–31.5 × 7.0–16.0 μm, pyriform or broadly clavate, smooth, hyaline, thin-walled. Lamellae trama interwoven, with hyphae 4.0–6.5 μm in diam, hyaline, dextrinoid, thin-walled. Stipitipellis a cutis composed of cylindrical hyphae, 4.0–9.5 μm wide, parallel, smooth. Caulocystidia absent. Clamp connections present.

##### Habitat.

Scattered on soil in broad-leaved forest or mixed coniferous and broad-leaved forests.

##### Additional specimens examined.

China • JiangXi Province, Jiangxi Agricultural University, 1 May 2019, collected by Jia-Yue Sun, HFJAU3367; 23 May 2023, collected by Lin-Gen Chen, Cheng-Feng Nie HFJAU4946; Hubei Province, Xingshan County, Yichang City, 23 July 2024, collected by Jun-Qing Yan, Lin-Gen Chen, Hong Chen, Ling Ding, HFJAU5635.

##### Note.

Based on molecular systematics and morphological analysis, *M.blandus* belongs to subg. Globularesser.Brunneospermi (Oliveira et al. 2020b; Oliveira et al. 2024), within this series, *M.blandus* is morphologically similar to *M.brunneospermus*, *M.fusicystidiosus*, and *M.macrocystidiosus*. However, *M.brunneospermus* has an irregularly wrinkled to rugulose reticulate pileus and larger cheilocystidia (30–57 × 4–13 μm) (Takahashi 1999); *M.fusicystidiosus* differs in the reddish ochre-brown pileus at the center, slightly paler towards margin and larger basidiospores (8.5–10 × 3.5–4.0 μm) (Antonín et al. 2010a); *M.macrocystidiosus* is distinguished from *M.blandus* by a light brown or grayish brown pileus, larger basidiospores (6.9–10.5 × 3.3–4.4 μm) and larger pleurocystidia (78.3–123.0 × 12.5–13.8 μm) (Kiyashko et al. 2014).

In addition, morphologically, among the known species of sect. Globulares, only *M.desjardinii* K. Das, Antonín & D. Chakr., *M.muramwyanensis* Antonín, *M.goossensiae* Beeli, and *M.phlebodiscus* Desjardin & E. Horak. share similar morphological characteristics with the new species *M.blandus*, including a hymenidermal pileipellis composed of *Globulares*-type cells, the basidiospores range in size from 5.0–8.0 × 2.0–4.0 μm, and have the well-developed pleurocystidia. However, *M.desjardinii* differs from *M.blandus* by a longer stipe (70–180 × 4–10 mm), grayish orange to apricot pileus when dry, pleurocystidia sometimes lageniform and possesses pileocystidia (Das et al. 2019); *M.muramwyanensis* differs by having a stipe white at apex, ochraceous to brownish-orange at base, pleurocystidia cylindrical, clavate, fusoid (Antonín 2003); *M.goossensiae* differs from *M.blandus* in forming a cream colored pileus with fuligineous or ochraceous brown centre, clavate, and rostrate pleurocystidia (Antonín 2007); *M.phlebodiscus* has a pale beige to tan colored pileus with reticulate wrinkles at center and only fusoid pleurocystidia, fusoid and mucronate cheilocystidia (Petrini et al. 1997).

#### ﻿ Marasmius xingshanensis

Taxon classificationFungiAgaricalesMarasmiaceae

J.Q. Yan & H. Chen sp. nov.

CF29FF77-103B-5DF8-9D12-295F580AF64A

858719

Fig. 3

##### Etymology.

“xingshanensis” refers to its type specimen originating from the Xingshan County of China.

**Figure 3.**
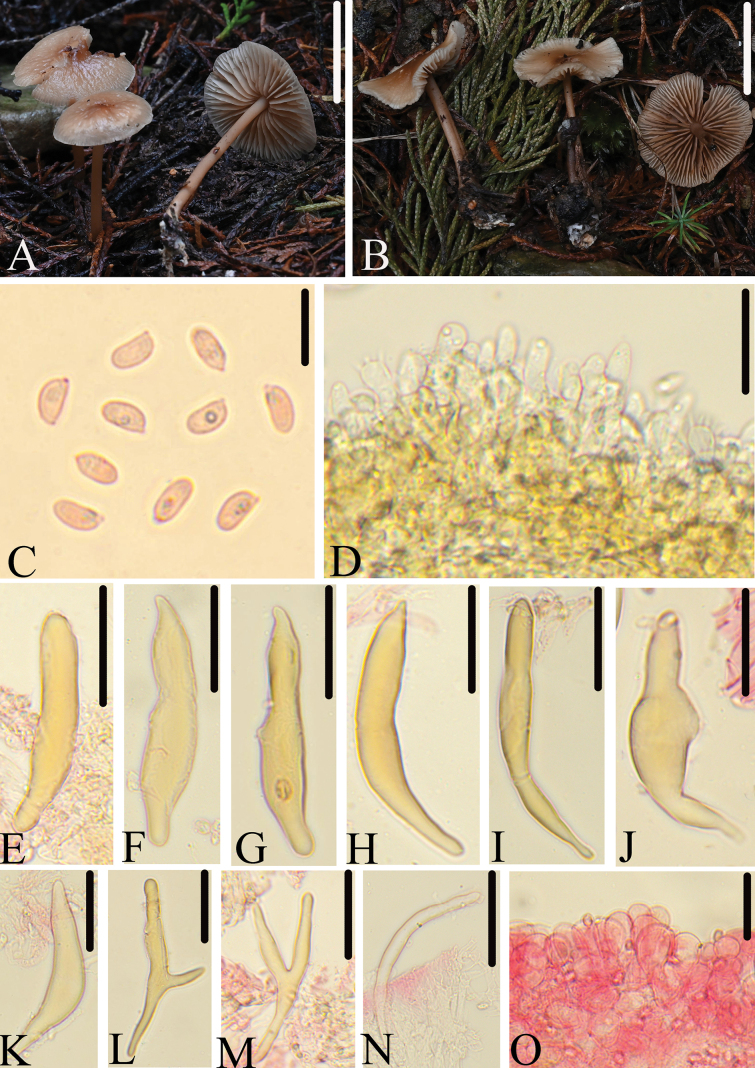
Morphological structures of *M.xingshanensis*. A, B. Basidiomata; C. Basidiospores; D. Lamellar edge; E–N. Pleurocystidia; O. Pileipellis. All microscopic structures were observed in 5% KOH, structures of C and E–O were stained by 1% Congo red. Scale bars: 20 mm (A, B); 10 μm (C); 30 μm (D–N); 20 μm (O).

##### Holotype.

China • Hubei Province, Xingshan County, Yichang City, 29 June 2024, collected by Jun-Qing Yan, Lin-Gen Chen, Hong Chen, Ling Ding, HFJAU5544.

##### Diagnosis.

*Marasmiusxingshanensis* is mainly characterized by the rather small basidiomata, pileus dark brown, reddish brown at center, slightly paler to white towards margin; lamellae rarely forked; basidiospores mainly shorter than 7.0 μm; well-developed pleurocystidia, up to 100 μm long, subcylindrical, ventricose, narrowly utriform, apex with obtuse or short papilla, the base is constricted into a curved long or short stipe; lamellae edge is composed of a large number of basidioles and rarely basidia; cheilocystidia absent. It differs from *M.riparius* Singer by having smaller spores which are shorter than 7 μm.

##### Description.

Pileus 15–30 mm, plano-convex to plane, center with slightly obtuse umbo, smooth, hygrophanous, dark brown (7F7), reddish brown (8D5–6) at center, slightly paler to white towards margin, striate up to 1/3 from the margin, drying out to white, with grayish orange at center. Context thin, white. Lamellae 2.0–3.0 mm broad, adnexed, ventricose, moderately close, grayish red (7B3), light brown (7D5), with 2–3 tiers of lamellules, rarely forked, edges even, white. Stipe 20–41 mm long, 1.5–3.0 mm thick, central, cylindric, equal, fibrous, hollow, grayish red (7B3), light brown (7D5), smooth, the base covered with white mycelium.

Basidiospores (5.0)5.5–7.0(7.5) × 2.2–3.5 μm (av = 6.3 × 2.9 μm), Q = (1.6)1.8–2.7(3.0), elongated-ellipsoid to cylindrical, slightly flattened on one side in profile, 2.3–3.5 μm broad, elongated-ellipsoid to cylindrical in face view, smooth, colorless, hyaline, inamyloid, thin-walled. Basidia 24.5–33.5 × 4.0–6.5 μm, clavate, 4-spored. Pleurocystidia (43.0)53.0–90.0(100.0) × (5.7)7.0–16.0 μm, variously, subcylindrical, ventricose, narrowly utriform, rarely branched, apex obtuse or with short papilla, the base is constricted into a curved long or short stipe, surface rarely with nodulose, smooth, slightly thick-walled, yellowish in 5% KOH. Lamellae edge is composed of a large number of basidioles and rarely basidia, cheilocystidia absent. Pileipellis a hymeniderm composed of cells 19.0–38.0 × 7.5–18.0 μm, pyriform or broadly clavate, smooth, hyaline, thin-walled. Lamellae trama interwoven, with hyphae 5.0–8.8 μm in diam, hyaline, dextrinoid, thin-walled. Stipitipellis a cutis composed of cylindrical hyphae 4.0–8.5 μm wide, parallel, smooth. Caulocystidia absent. Clamp connections present.

##### Habitat.

Scattered on soil in broad-leaved forest or mixed coniferous and broad-leaved forests.

##### Additional specimens examined.

China • Hubei Province, Xingshan County, Yichang City, 29 June 2024, collected by Jun-Qing Yan, Lin-Gen Chen, Hong Chen, Ling Ding, HFJAU5344; 4 July 2024 collected by Jun-Qing Yan, Lin-Gen Chen, Hong Chen, Ling Ding, HFJAU5540.

##### Note.

Based on molecular systematics and morphological analysis, *M.xingshanensis* belongs to subg. Globularesser.Brunneospermi (Oliveira et al. 2020b; Oliveira et al. 2024). Within this series, only *M.magnus* lacks cheilocystidia. However, *M.magnus* has a larger pileus (31–122 mm), fulvous to rusty orange in color, a longer stipe (70–94 mm), and a pileipellis composed of *Siccus*-type broom cells (Magnago et al. 2016).

Morphologically, among the known species of sect. Globulares, only *M.riparius* and *M.ochraceus* Berk. & Broome. share similar morphological characteristics with the new species *M.xingshanensis*, including a hymenidermal pileipellis composed of *Globulares*-type cells, well-developed pleurocystidia, and lack of cheilocystidia. However, *M.riparius* has a cinnamon pileus, with larger basidiospores (8.2–10 × 4.8–5.5 μm), smaller pleurocystidia (40–44 × 8–9 μm) (Singer and Digilio 1952); *M.ochraceus* has a larger pileus (30–80 mm), a longer stipe (60–110 mm) and smaller pleurocystidia (30–35 × 6–8 μm) (Berkeley and Broome 1873).

In addition, morphologically, *M.pallidibrunneus* J.S. Oliveira, and *M.pinicola* Jing Si, S.H. He & Hai J. Li have the aspect of *M.xingshanensis* with similarly basidioma size and colored pileus. However, *M.pallidibrunneus* has basidiospores up to 9 μm long, and presence of cheilocystidia (Oliveira et al. 2020b); *M.pinicola* has smaller pleurocystidia (26–46 × 5–12 μm), and presence of cheilocystidia (Li et al. 2023).

#### ﻿ Marasmius vulgaris

Taxon classificationFungiAgaricalesMarasmiaceae

J.Q. Yan & H. Chen sp. nov.

73AAFB58-BA6E-5874-BD3D-ED062BDA8D10

858720

Fig. 4

##### Etymology.

“vulgaris” means “common” or “usual”, refers to the fact that many known species in this genus share similar macroscopic characteristics with the new species.

**Figure 4.**
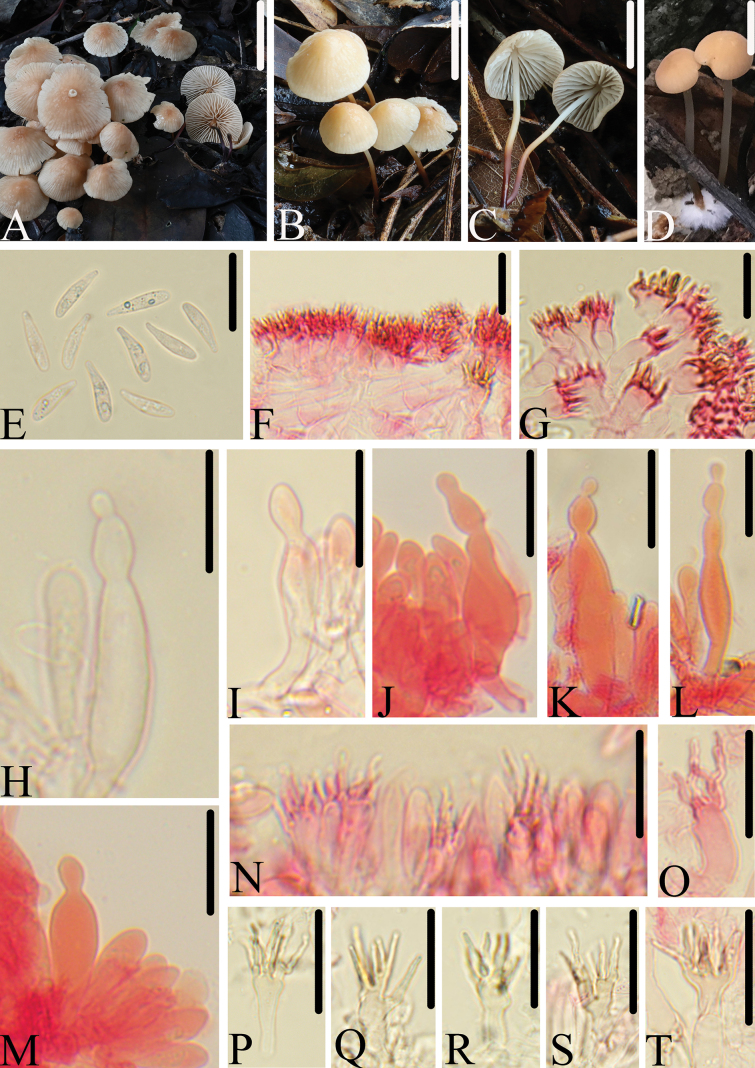
Morphological structures of *M.vulgaris*. A–D. Basidiomata; E. Basidiospores; F, G. Pileipellis; H–M. Pleurocystidia; N–T. Cheilocystidia. All microscopic structures were observed in 5% KOH, structures of E–O and S–T were stained by 1% Congo red. Scale bars: 20 mm (A–D); 20 μm (E–T).

##### Holotype.

China • ZheJiang Province, QingTian County, LiShui City, collected by Qin Na, Bin-Rong Ke, Zhi-Heng Zeng, 6 August 2021, HFJAU2875.

##### Diagnosis.

*Marasmiusvulgaris* is mainly characterized by the rather small basidiomata, having a brownish orange, grayish orange pileus; with 3–5 tiers of lamellules; stipe white at apex, gradually darkening from the apex to the base, reddish brown towards base; basidiospores 13.5–16.0 × 3.0–4.0 μm, pleurocystidia abundant, narrowly fusiform, lageniform, which frequently have capitate, papillate, or constricted moniliform structures at apices; pileipellis and cheilocystidia in the form of broom-cells of the *Siccus*-type. It differs from M.confertusvar.tenuicystidiatus Antonín by having bigger spores, which are up to 16.0 μm in length.

##### Description.

Pileus 10–30 mm, convex when young, then broadly conical to plane, smooth, with or without slightly obtuse umbo, brownish orange (7C4–5), grayish orange (5B3) at center, slightly paler to white towards margin, striate up to 2/3 from the margin. Context thin. Lamellae 2.0–3.0 mm broad, adnexed, ventricose, moderately close, white, with 3–5 tiers of lamellules, edges even, concolorous. Stipe 25–65 mm long, 1.5–2.0 mm thick, central, cylindric, rarely twisted, equal, fibrous, hollow, white at apex, gradually darkening from the apex to the base, reddish brown towards base, smooth, and the base covered with white mycelium.

Basidiospores (12.5)13.5–16.0(17.0) × 3.0–4.0(4.5) μm (av = 14.8 × 3.8 μm), Q = (3.0)3.3–5.0(5.2), clavate or fusoid-clavate, often curved in profile, 3.0–4.0 μm broad, clavate in face view, smooth, colorless, hyaline, inamyloid, thin-walled. Basidia 25.0–35.0 × 4.5–6.5 μm, clavate, 4-spored. Pleurocystidia 33.0–58.0 × 5.0–11.0 μm, narrowly fusiform, lageniform, frequently capitate, papillate, or constricted moniliform structures at apices. Cheilocystidia abundant, in form of *Siccus*-type broom cells, main body 13.0–26.0 × 4.0–11.0 μm, cylindrical to clavate, hyaline, thin-walled; apical setulae 4.0–13.0 × 1.0–2.0 μm, cylindrical to conical, subacute, yellow to pale yellow, thick-walled. Pileipellis hymeniform, mottled, composed of *Siccus*-type broom cells; main body 14.0–27.0 × 5.5–9.0 μm, clavate or pyriform, hyaline to pale yellow, thin- to thick-walled; apical setulae 4.0–10.5 × 0.8–1.5 μm, crowded, cylindrical, subacute, thick-walled. Lamellae trama interwoven, with hyphae 7.0–10.0 μm in diam, hyaline, dextrinoid, thin-walled. Stipitipellis a cutis composed of cylindrical hyphae 4.5–11.0 μm wide, parallel, smooth. Caulocystidia absent. Clamp connections present.

##### Habitat.

Scattered on soil in broad-leaved forest or mixed coniferous and broad-leaved forests.

##### Additional specimens examined.

China • JiangXi Province, Lushan National Nature Reserve, collected by Jing-Cheng Wu, Hong-Zhao Pan, 30 June 2019, HFJAU0904; ZheJiang Province, SuiChang County, collected by Jun-Qing Yan, Yan-Liu Chen, 12 July 2020, HFJAU1901; collected by Yu-Peng Ge, Bin-Rong Ke, Zhi-Heng Zeng, 14 July 2020, HFJAU1976; ZheJiang Province, QingTian County, LiShui City, collected by Jun-Qing Yan, Ze-Wei Liu, 5 August 2021, HFJAU2719, HFJAU2748.

##### Note.

Based on molecular and morphological evidence, *M.vulgaris* belongs to subg. Globularesser.Conferti (Oliveira et al. 2020b; Oliveira et al. 2024). Within this series, *M.vulgaris* is morphologically similar to M.confertusvar.tenuicystidiatus, *M.bondoi* Wannathes, Desjardin & Lumyong, and *M.graminipes*. However, M.confertusvar.tenuicystidiatus has smaller basidiospores (10–14 × 4.5–5.7 μm), a pruinose stipe apex, sometimes rostrate pleurocystidia (Antonín et al. 2011); *M.bondoi* has a pruinose pileus surface, pleurocystidia with lobed apices, and the absence of papillate structures (Wannathes et al. 2009a); *M.graminipes* has larger basidiospores (18–21 × 4 μm) and the presence of caulocystidia (Yang et al. 2013).

Morphologically, *M.eyssartieri* Antonín & Buyck and *M.subtangerinus* Antonín, Ryoo & H.D. Shin share similar morphological characteristics with the new species *M.vulgaris*, including basidiospores measuring 13–16 μm in length, the presence of pleurocystidia and pileipellis and cheilocystidia in the form of broom cells of the *Siccus*-type. However, *M.eyssartieri* differs in having a smaller pileus (5 mm), 0–1 tier of lamellulae, and ‌clavate to occasionally rostrate pleurocystidia (Antonín and Buyck 2006), while *M.subtangerinus* has 0–1 tier of lamellulae and clavate to rostrate pleurocystidia (Antonín et al. 2011).

In addition, morphologically, *M.rongklaensis* Wannathes, *M.subabundans* Chun Y. Deng & T.H. Lim, and *M.pseudoconfertus* T.H. Li & Chun Y. Deng. have the aspect of *M.vulgaris* with similarly colored pileus and stipe. However, *M.rongklaensis* has smaller basidiospores (9–11 × 4.5–5.5 μm) and presence of cheilosetae and pileosetae (Wannathes et al. 2019); *M.subabundans* has ‌smaller basidiospores (7–9 × 3–4.5 μm) and absence of pleurocystidia (Deng et al. 2012); *M.pseudoconfertus* has the slightly wider basidiospores (4–5 μm) and absence of pleurocystidia (Deng et al. 2011).

#### ﻿ Marasmius subpurpureostriatus

Taxon classificationFungiAgaricalesMarasmiaceae

J.Q. Yan & H. Chen sp. nov.

AB28C288-7542-5C1C-AF64-A25B877FD223

858721

Fig. 5

##### Etymology.

“subpurpureostriatus” refers to its macroscopic morphology, which is similar to that of *Marasmiuspurpureostriatus*.

**Figure 5.**
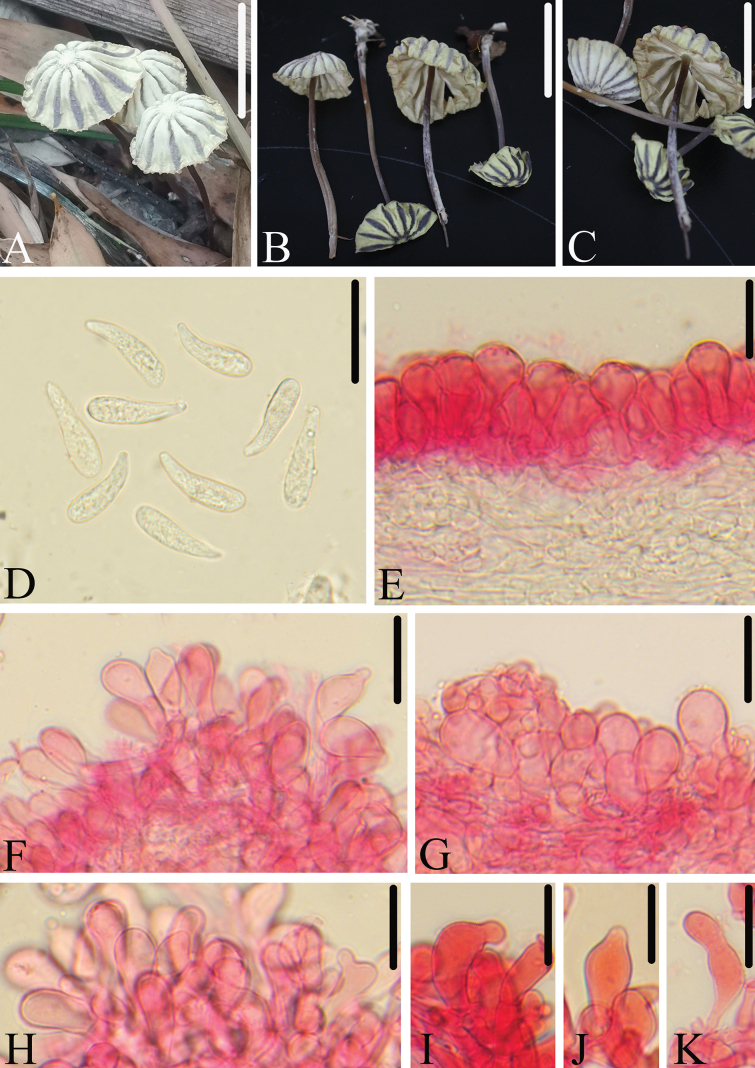
Morphological structures of *M.subpurpureostriatus*. A–C. Basidiomata; D. Basidiospores; E. Pileipellis; F–K. Cheilocystidia. All microscopic structures were observed in 5% KOH, structures of E–K were stained by 1% Congo red. Scale bars: 30 mm (A–C); 20 μm (D–K).

##### Holotype.

China • JiangXi Province, Nanchang Botanical Garden, 26 April 2023, collected by Xiao-Lin Yuan, HFJAU4740.

##### Diagnosis.

*Marasmiussubpurpureostriatus* is mainly characterized by the rather small basidiomata, grayish-green pileus with deep violet sulcate; the stipe surface is densely pruinose; basidiospores clavate to fusoid-clavate, 17.5–22.0 × 4.0–5.5 μm; pleurocystidia absent; cheilocystidia clavate to broadly clavate, occasionally appearing subfusiform, apex obtuse, rarely with short papilla. It differs from *M.purpureostriatus* by having smaller spores, which are shorter than 22 μm.

##### Description.

Pileus 20–32 mm, plano-convex to plane, with obtuse umbo at center, smooth, with dark sulcate up to center from the margin, grayish-green (28B4), light green (28A4–5) sulcate dark violet (18F4). Context thin. Lamellae 1.0–2.5 mm broad, adnexed, ventricose, distant, with 0–1 tier of lamellulae, yellowish gray (4B2) with white, edges even, concolorous. Stipe 53–70 mm long, 1.5–3.0 mm thick, central, cylindrical with a subbulbous base, pruinose, hollow, apex dark violet (15F5), gradually paler toward the base, base reddish brown (8E4). The base covered by white mycelium.

Basidiospores (16.5)17.5–22.0(23.5) × 4.0–5.5(6.0) μm (av = 20.0 × 5.0 μm), Q = (3.0)3.5–4.5(5.0), clavate to fusoid-clavate, often curved in profile, 4.0–6.5 μm broad, clavate in face view, smooth, colorless, hyaline, inamyloid, thin-walled. Basidia 40.0–50.0 × 8.0–14.0 μm, clavate, 4-spored. Pleurocystidia absent. Cheilocystidia 13.0–31.0 × 6.0–13.0 μm, irregularly clavate to broadly clavate, rarely subfusiform, apex obtuse, rarely with short papilla​, smooth, thin-walled. Pileipellis a hymeniderm composed of cells 16.5–34.0 × 9.0–16.0 μm, pyriform or broadly clavate, smooth, hyaline, thin-walled. Lamellae trama interwoven, with hyphae 4.0–10.0 μm in diam, hyaline, dextrinoid, thin-walled, non-gelatinous. Stipitipellis a cutis composed of cylindrical hyphae 4.0–7.5 μm wide, parallel, smooth. Caulocystidia absent. Clamp connections present.

##### Habitat.

Scattered on soil in broad-leaved forest.

##### Note.

Based on molecular and morphological evidence, *M.subpurpureostriatus* belongs to subg. Globularesser.Purpureostriati (Oliveira et al. 2020b; Oliveira et al. 2024). Within this series, *M.subpurpureostriatus* is morphologically similar to *M.purpureostriatus*, *M.pseudopurpureostriatus* Wannathes, Desjardin & Lumyong, and *M.albopurpureus* T.H. Li & C.Q. Wang. However, *M.purpureostriatus* is distinguished by a long and narrow stipe (52–103 × 0.5–1.5 mm), larger basidiospores (up to 28 μm long), and cheilocystidia that are exclusively cylindrical, broadly clavate, or pyriform (Desjardin and Horak 1997; Wannathes et al. 2009a). *M.pseudopurpureostriatus* has a ‌glabrous stipe surface, larger basidiospores (up to 25.0 μm long), and cheilocystidia restricted to clavate or broadly clavate forms (Wannathes et al. 2009a); *M.albopurpureus* is distinguished by a strongly rugulose to sulcate pileus (white to purple), ‌‌cream to purple lamellae, and cheilocystidia that are solely clavate to broadly clavate (Wang et al. 2015).

In addition, morphologically, only *M.bekolacongoli* Beeli and *M.violaceoides* Antonín share similar morphological characteristics with the new species *M.subpurpureostriatus*, including a hymenidermal pileipellis composed of *Globulares*-type cells, the basidiospores measuring 16.0–24.0 μm in length, and absence of pleurocystidia. However, *M.bekolacongoli* has a larger pileus (30–67 mm) with a yellow colored or tinged striae, and a much longer stipe (50–150 mm) with pale yellow and light brown downward color (Beeli 1928; Douanla-Meli and Langer 2008; Antonín et al. 2010a); *M.violaceoides* exhibits a distinctly campanulate, violaceous pileus, a very long and glabrous stipe (110–125 mm) (Antonín 2004).

## ﻿Discussion

This study strongly supports the phylogenetic findings of Oliveira et al. (2024). In the phylogenetic tree (Fig. 1), the four new species were each assigned to clade /brunneospermus, /confertus and /purpureostriatus (Oliveira et al. 2020b; Oliveira et al. 2024). Specifically, Oliveira et al. (2020b) proposed the series *Brunneospermi* based on the clade /brunneospermus, which is characterized by a hygrophanous pileus; 2–3 (rarely 4–5) tiers of lamellulae; present elongate pleurocystidia; pileipellis mostly composed of *Globulares*-type cells, rarely composed of *Siccus*-type cells; the series *Conferti* based on the clade /confertus, which is characterized by an orange to ochraceous, or olivaceous pileus; 2–5 (or more) tiers of lamellulae; present versiform pleurocystidia; pileipellis consisting of *Siccus*-type cells; and the series *Purpureostriati* based on the clade /purpureostriatus, which is characterized by a markedly sulcate pileus; distant lamellae, 0–2 tiers of lamellulae; absent pleurocystidia; pileipellis consisting only of *Globulares*-type cells (Oliveira et al. 2020b). Four new species also respectively conform to these characteristics.

In the phylogenetic tree (Fig. 1), *M.blandus* groups together with *M.brunneospermus* and *M.fusicystidiosus*; *M.vulgaris* is closely related to *M.graminipes*; and *M.subpurpureostriatus* is closely related to *M.purpureostriatus*. The distinctions among these species have been discussed in the section of note. In addition, *M.xingshanensis* groups together with *M.macrocystidiosus*. However, *M.macrocystidiosus* can be distinguished from *M.xingshanensis* by having larger basidiospores (6.9–10.5 × 3.3–4.4 μm) and presence of cheilocystidia (Kiyashko et al. 2014).

In conclusion, the phylogenetic tree strongly supports new species distinct clade, suggesting that *Marasmius* may contain undiscovered biodiversity. Further studies are needed to elucidate its evolutionary history and ecological roles, thereby enhancing our understanding.

### ﻿Key to related species

**Table:** 

1	Pileipellis is composed of *Siccus*-type cells	**2**
–	Pileipellis is composed of *Globulares*-type cells	**11**
2	Cheilosetae and pileosetae present	***M.rongklaensis***
–	Cheilosetae and pileosetae absent	**3**
3	Basidiospores mainly shorter than 10.0 μm	**4**
–	Basidiospores mainly longer than 10.0 μm	**5**
4	Cheilocystidia present	***M.subabundans***
–	Cheilocystidia absent	***M.magnus***
5	Pleurocystidia absent	***M.pseudoconfertus***
–	Pleurocystidia present	**6**
6	Basidiospores mainly longer than 18.0 μm	***M.graminipes***
–	Basidiospores mainly shorter than 18.0 μm	**7**
7	Pileus up to 65 mm wide, stipe with pruinose at apex	**M.confertusvar.tenuicystidiatus**
–	Pileus mainly < 30 mm in diameter	**8**
8	Lamellules with 0–1 tier	**9**
–	Lamellules with 2–5 tier	**10**
9	Lamellae pale yellowish white to pale cream	***M.subtangerinus***
–	Lamellae brown, pubescent edge	***M.eyssartieri***
10	Pileus with pruinose, pleurocystidia with lobed apices, and the absence of papillate structures	***M.bondoi***
–	Pileus glabrous, pleurocystidia apices with capitate, papillate and constricted into moniliform structures	***M.vulgaris***
11	Basidiospores mainly longer than 10.0 μm	**12**
–	Basidiospores mainly shorter than 10.0 μm	**17**
12	Pileus grayish green and light green, sulcate dark violet	***M.subpurpureostriatus***
–	Pileus brown to red or violet	**13**
13	Pileus with a yellow colored or tinged striae and stipe with pale yellow and light brown downward color	***M.bekolacongoli***
–	Not as above	**14**
14	Basidiospores 15.5–22.0 μm long, stipe 110–125 × 2.5–3.5 mm	***M.violaceoides***
–	Basidiospores mainly longer than 22.0 μm, stipe mainly < 100 mm long	**15**
15	Pileus larger, up to 38 mm, stipe apex grayish magenta	***M.pseudopurpureostriatus***
–	Pileus rather small, only up to 20 mm, stipe apex violet	**16**
16	Pileus with dark violet ridges, stipe 0.5–1.5 mm thick, basidiospores up to 28 μm long	***M.purpureostriatus***
–	Pileus with white to violet white ridges, stipe 1.5–3 mm thick, basidiospores shorter than 25 μm long	***M.albopurpureus***
17	Cheilocystidia present	**18**
–	Cheilocystidia absent	**27**
18	Basidiospores mainly > 3.5 μm width	**19**
–	Basidiospores mainly < 3.5 μm width	**23**
19	Pleurocystidia mainly shorter than 50 μm	***M.pinicola***
–	Pleurocystidia mainly longer than 50 μm	**20**
20	Cheilocystidia mainly shorter than 30 μm	**21**
–	Cheilocystidia mainly longer than 30 μm	**22**
21	Basidiospores 8.5–10.0 × 3.5–4.0 μm, stipe 110 × 3 mm	***M.fusicystidiosus***
–	Basidiospores 6.2–7.9 × 3.5–4.2 μm, stipe 50–70 × 3–6 mm	***M.goossensiae***
22	Stipe grayish orange	***M.macrocystidiosus***
–	Stipe white or grayish white	***M.pallidibrunneus***
23	Pleurocystidia subfusiform, narrowly utriform, apex with short or long papilla, surface rarely with nodulose	***M.blandus***
–	Not as above	**24**
24	Basidiospores mainly longer than 6.5 μm	**25**
–	Basidiospores mainly shorter than 6.5 μm	**26**
25	Stipe pale ochraceous towards the base or pale whitish-brown or grayish-brown, pileocystidia absent	***M.brunneospermus***
–	Stipe yellowish white, turning brownish orange to light brown on bruising, pileocystidia present	***M.desjardinii***
26	Pleurocystidia only fusoid	***M.phlebodiscus***
–	Pleurocystidia cylindrical, clavate, fusoid, often subrostrate	***M.muramwyanensis***
27	Basidiospores mainly longer than 8.0 μm	***M.riparius***
–	Basidiospores mainly shorter than 7.0 μm	**28**
28	Pileus mainly > 30 mm in diameter, stipe 60–110 × 4–7 μm, pileocystidia 30–35 × 6–8 μm	***M.ochraceus***
–	Pileus mainly < 30 mm in diameter, stipe 20–41 × 1.5–3 μm, pileocystidia 53–90 × 7–16 μm	***M.xingshanensis***

## Supplementary Material

XML Treatment for Marasmius blandus

XML Treatment for Marasmius xingshanensis

XML Treatment for Marasmius vulgaris

XML Treatment for Marasmius subpurpureostriatus

## ﻿Additional information

### Use of AI

No use of AI was reported.

### Author ORCIDs

Hong Chen https://orcid.org/0009-0004-0107-3962

Yu-Qin Xu https://orcid.org/0009-0002-0735-8323

Hui Zeng https://orcid.org/0000-0003-2025-844X

Ya-Ping Hu https://orcid.org/0000-0003-1242-1139

Sheng-Nan Wang https://orcid.org/0000-0003-0648-271X

Jun-Qing Yan https://orcid.org/0000-0003-1128-5171

### Data availability

All of the data that support the findings of this study are available in the main text.
